# Whole person medicine: Psychosocial issues in primary care

**DOI:** 10.4102/phcfm.v8i1.1328

**Published:** 2016-11-16

**Authors:** Robert Mash

**Affiliations:** 1 Division of Family Medicine and Primary Care, Department of Interdisciplinary Health Sciences, Stellenbosch University, South Africa

Organising health services around the needs of people rather than designated conditions or diseases is one of the key reforms proposed in the 2008 World Health Report ‘Primary health care: Now more than ever’.^1^ Many health systems have subsequently espoused the centrality of person-centeredness. For example, in South Africa the health system in the Western Cape states that ‘The crux of a re-imagined future in 2030 is the focus on person-centredness’.^2^

The concept of person-centredness is central to the competencies expected of medical generalists. Prof. Howe in her report ‘Medical generalism: Why whole person medicine matters’^3^ defined generalism as

1. seeing the person as a whole and in the context of his or her family and wider social environment;
2. using this perspective as part of the clinical method and therapeutic approach to all clinical encounters;
3. being able to deal with undifferentiated illness and the widest range of patients and conditions;
4. in the context of general practice, taking continuity of responsibility for people’s care across many disease episodes and over time; …
5. coordinating his or her care as needed across organisations within and between health and social care.

In the African context, person-centredness may need to adapt to the importance of the family network in more communal decision-making.^4^

In this context, primary care may be offered by a variety of medical generalists that include clinical nurse practitioners, mid-level doctors (e.g. clinical officers or clinical associates) and doctors. The question arises as to whether our medical generalists have the capacity to offer a service that is person-centred.

The capacity of primary care providers to be person-centred may depend not only on their capability as practitioners, but also on their organisational context and environment. Several studies have demonstrated that the organisational culture is often unsupportive and incongruent with values that promote person-centredness.^5,6^ Health workers report a culture that is characterised by a lack of information sharing, blame, manipulation, hierarchy, bureaucracy, confusion and working in isolation. If person-centredness is a key attribute of primary health care, then it should apply to both staff and patients as a core characteristic of the health system. Unfortunately, high levels of burnout and depression have been reported in our primary care providers.^7^ Depersonalisation and withdrawal are the unfortunate consequences, which makes the realisation of person-centredness more difficult.

The patient’s overall experience of the health facility may also be critical in creating a culture of person-centredness. Issues such as long waiting times, lack of cleanliness, poor staff attitudes, lack of information, inadvertent breaking of confidentiality and perceived unfairness or discrimination may erode the patient’s experience long before they see their primary care provider.^8^

The capability of primary care providers, however, to behave as competent generalists who deliver person-centred care is also an issue. The issues of capability may go deeper than training in specific skills to the underlying paradigm and the way in which we conceptualise ourselves as practitioners. Doctors have been criticised for seeing themselves as natural scientists who regard the person as a scientific problem to be solved rather than a unique and complex human being to be understood.^9^ Nurses have been criticised for seeing primary care as a series of tasks to be performed according to a prescribed guideline rather than a responsibility to care for a whole person.^10^

There are of course specific tasks in the consultation that can be identified and taught to enable the competency of person-centredness.^11^ A fundamental task is understanding why the person has consulted you today as well as working out what is the diagnosis.^12^ This person is likely to have consulted others in their family, community or traditional healers and to have a variety of explanations for their illness. They may be consulting because of the severity of their symptoms, because of the concern for what these symptoms might mean, because of underlying problems of living, because they need some administrative help or in order to prevent a disease. Responding to the real reason for encounter makes the consultation more efficient and person-centred. In the first half of the consultation, therefore, generalists should routinely encourage the person to tell their story, gather information about the person’s expectations, concerns and ideas, understand something of the person’s unique context, in addition to seeking out diagnostic information.^11^ In the second half of the consultation, it becomes important to consider the patient’s preferences, ideas, opinions or goals when agreeing together on an appropriate plan of action.^11^ Understanding what is important to the patient may help to decide what to focus on, especially when there is multimorbidity and a plethora of recommendations from guidelines.^13^

Underlying the accomplishment of these tasks would be skills in asking open as well as closed questions, affirming and acknowledging the patient, active listening with the use of reflections and summaries and an ability to exchange information effectively.^14^

Certain common conditions in primary care require a holistic and person-centred approach in order to recognise them. The ability to recognise them could be seen as an indicator of the person-centredness of a health service. Mental disorders are known to be present in up to a quarter of primary care consultations with depression, anxiety, harmful alcohol use, sleep problems, chronic tiredness and unexplained somatic complaints being the most common.^15^ Poor recognition by primary care providers has been linked to a failure to generate psychological hypotheses in decision-making, poor communication skills, language and cultural barriers, lack of continuity of care, short consultations, beliefs of the provider and a lack of knowledge and training.^15^ In a recent primary care morbidity survey in South Africa, depression and anxiety were almost never recognised.^16^ Intimate partner violence is another common problem that challenges primary care providers to be holistic and person-centred.^17^ A holistic approach requires attention to clinical, psychological, legal, forensic and social issues. Again a recent study in South Africa demonstrated that less than 10% of women attending primary care over a 2-year period were identified as suffering from intimate partner violence.^18^

What then can be done to help us reform our primary health care services to be more person-centred? I would suggest the following:

**TABLE 1:** Comparing paradigms in the consultation.

Biological medicine	Whole person medicine
Single agenda – make a diagnosis	Dual agenda – understand the reason for encounter and make a diagnosis
Avoids complexity	Comfortable with complexity
Wants to fix a problem	Wants to help a person
Directive	Guiding
Focus on disease	Focus on wellness
Task-orientated consultations	Person-centred consultation

- Change our underlying paradigm from biological to whole person medicine as shown in Table 1
- Transform organisational culture to be congruent with person-centredness for staff and patients
- Improve the patient’s overall experience of the health facility
- Train all our generalists in how to consult in a person-centred approach with the requisite communication skills and within a primary care system designed for access, continuity, co-ordination and comprehensiveness.^19^

The discipline of family medicine can make a significant contribution as whole person medicine and person-centredness is already embraced as a core characteristic.^20^ Family medicine should be involved in the training of all primary care providers to embody person-centredness in all generalists. Family physicians can also assist with transforming organisational culture^21,22^ and the patient’s experience^23^ as they are senior clinicians with a responsibility for clinical governance within the health system.
